# Curcumin for the treatment of pituitary adenomas: the potential of a single agent for multifaceted therapeutic effects

**DOI:** 10.3389/fendo.2025.1648521

**Published:** 2025-10-03

**Authors:** Zisheng Yan, Chen Liang, Fujia Nian, Gang Peng, Yuntao Li, Jingyu Guan, Ruihan Pan, Gaochao Song

**Affiliations:** ^1^ Department of Neurosurgery, The Second Affiliated Hospital of Nanjing Medical University, Nanjing Medical University, Nanjing, China; ^2^ Department of Neurology, The Second Affiliated Hospital of Nanjing Medical University, Nanjing Medical University, Nanjing, China; ^3^ Department of General Medicine, The Second Affiliated Hospital of Nanjing Medical University, Nanjing Medical University, Nanjing, China

**Keywords:** curcumin, pituitary adenoma, hormone disorder, metabolic disorder, nanovesicle

## Abstract

Pituitary adenomas (PAs), accounting for 10–15% of intracranial tumors, cause significant morbidity through endocrine dysfunction and mass effects. While current treatments (surgery, pharmacotherapy, radiation) face challenges such as drug resistance, recurrence, and metabolic complications, curcumin emerges as a promising multi-target agent for PA management. This review synthesizes evidence on curcumin’s dual roles: suppressing tumor progression and ameliorating hormone-driven metabolic disorders.

**Antitumor mechanisms:**

Curcumin inhibits PA proliferation by modulating cell cycle proteins, inducing apoptosis via pro-apoptotic protein upregulation and anti-apoptotic suppression. It targets key pathways like NF-κB, reducing VEGF/HIF-1α-driven angiogenesis and MMP-9-mediated invasion. Synergistic effects enhance existing therapies: low-dose curcumin potentiates bromocriptine in prolactinomas by regulating ERK/EGR1 and AKT/GSK-3β, while in aggressive PAs, it may overcome temozolomide resistance by downregulating DNA repair enzymes.

**Metabolic regulation:**

Beyond antitumor effects, curcumin mitigates hormone-induced metabolic dysregulation. It suppresses excess ACTH, GH, and prolactin secretion in functional PAs. For GH adenomas, curcumin improves insulin resistance by activating AMPK, enhancing skeletal muscle glucose uptake, and suppressing hepatic gluconeogenesis. It also reduces inflammatory cytokines and oxidative stress, protecting against cortisol-induced glycometabolic dysfunction and PRL/GH-mediated bone loss via RANKL/OPG pathway modulation.

**Conclusion:**

Curcumin’s ability to concurrently target tumor growth, hormone hypersecretion, and metabolic complications positions it as a unique “one drug, multiple effects” candidate. Future research must prioritize PA-specific mechanistic studies and advanced delivery systems to realize its clinical potential.

## 1 Introduction

PAs are tumors that originate in the pituitary gland and currently account for 10-15% of intracranial tumors, including functioning pituitary adenomas (FPAs, approximately 70%) as well as non-functioning pituitary adenomas (NFPAs, approximately 30%) (1). NFPAs mainly cause locoregional effects in the saddle region, whereas FPAs can additionally produce excessive amounts of pituitary hormones, leading to pathophysiological changes that result in hormonal imbalances in the human body, such as prolactin adenoma that may lead to male dysfunction, and growth hormone (GH) adenoma that may induce acromegaly, which can lead to a range of metabolic disorders (2). Although the majority of PAs are benign and manageable with a combination of surgery, pharmacotherapy, and radiation (3). treatment challenges remain. For example, dopamine agonists like cabergoline and bromocriptine, the first-line therapy for prolactinomas, exhibit unsatisfactory efficacy in 10-30% of patients due to resistance or intolerance (4). There is a lot of research on targeted drugs that may potentially act on PAs, but fewer types are available to put into clinical trials at this stage (5). Similarly, there is still a high recurrence rate of surgical treatment of aggressive PAs and the risk of hypopituitarism associated with radiation therapy (6).

Curcumin is a naturally occurring polyphenolic compound extracted from the rhizome of the plant Curcuma longa, whose structure consists of two benzene rings connected by a β-diketone bridge, which confers on it a unique antioxidant and electron-donor capacity (7). Curcumin has been used for thousands of years in some traditional medicines, mainly for anti-inflammatory, antioxidant and anti-infective treatments (7). In previous studies, curcumin has demonstrated positive effects in the treatment of a variety of tumors (8, 9). Recently, it has been suggested in several studies that curcumin may play a role in the growth, autophagy, and drug resistance of PAs (10, 11), thereby exerting an anti-tumor effect. Further, curcumin has been studied in numerous endocrine studies for its potential role in improving glucose metabolism, regulating lipid metabolism, and as an anti-inflammatory and antioxidant (12, 13). These features give curcumin a unique advantage in the treatment of PAs, as it may target multiple aspects of proliferation, hormone secretion, and metabolic disorders simultaneously, thus compensating for the single-acting limitations of existing drugs (e.g., dopamine agonists, somatostatin analogs). Two databases, PubMed and Web of Science, were used for this literature screening. Titles were screened for the terms “pituitary adenoma” and “curcumin”, and relevant articles including basic research, clinical research, and reviews were retained.

Molecular mechanism of curcumin against pituitary adenomas

1. Inhibition of tumor cell proliferation and induction of apoptosis

Previous studies in other tumor cells have shown that curcumin has a corresponding antitumor potential (8). Inhibition of cell proliferation and induction of apoptosis are considered to be one of the main mechanisms by which curcumin exerts its anticancer effects. On the one hand, curcumin enhances the expression of pro-apoptotic proteins such as Bax, PUMA, Noxa, Bak and Bim, or apoptosis receptors such as TRAIL-R1/DR4 and TRAIL-R2/DR5 (14, 15); on the other hand, curcumin reduces the expression of anti-apoptotic proteins such as Bcl-2, Bcl-XL, survin, and XIAP(**Table 1**). Further, curcumin can directly induce apoptosis (20). or directly inhibit cell proliferation by arresting the cell cycle in the G2/M phase (21). As early as 2002, Mukhopadhy et al. showed that curcumin can regulate the levels of cell cycle protein-dependent kinases (CDK) and cell cycle proteins by increasing the expression of CDK inhibitors (22).

**Table 1:** Mechanism of curcumin in the treatment of pituitary adenomas.

Effect	Mechanism	Type	Reference
anti-proliferative	Induces G2/M phase cell cycle arrest; decreases Cyclin D1, CDK4 expression; inhibits p-Rb (Ser780) phosphorylation	*In vitro* and *in vivo*	(16, 17)
	Inhibition of NF-κB activity; down-regulation of Bcl-xL expression	*In vitro*	(18)
	Upregulates miR-206 expression; inhibits Akt/mTOR pathway; promotes autophagy	*In vitro*	(10)
	Inhibition of HIF-1α synthesis; reduction of VEGFA expression	*In vitro* and *in vivo*	(19)
pro-apoptotic	Induction of early apoptosis; reduction of Bcl-2	*In vitro*	(16, 17)
	Induces mitochondrial membrane depolarization; increases PARP cleavage	*In vitro*	(18)
	Promoting autophagy through miR-206	*In vitro*	(10)

And in PAs, a 2008 study by Miller et al. noted that curcumin inhibited the colony-forming ability of PA cells (17). The growth-inhibitory effect of curcumin was accompanied by decreased expression of cyclin D3 and ser 780 phosphorylation of retinoblastoma protein. However, at the time, this study did not explore the molecular mechanism of this effect any further. Subsequent studies have suggested that curcumin may affect the proliferation of PA cells by modulating some of the classical signaling pathways, and curcumin has been shown to be a potent inhibitor of the NF-κB pathway, which has been implicated in the growth of colon, breast, and prostate cancers, among others, in the literature. For instance, one study demonstrated that curcumin induced growth inhibition in mouse AtT20 cells by suppressing NF-κB activity (23). In addition, curcumin down-regulated the anti-apoptotic protein Bcl-xL, depolarizing the mitochondrial membrane and leading to apoptotic cell death (18). Another study in the same year similarly demonstrated that curcumin leads to decreased expression of NF-κB in other types of PA cells (24). And this study showed that curcumin-induced apoptosis involves inhibition of Bcl-2, stimulation of cleaved caspase-3, and induction of DNA fragmentation.

## 2 Anti-angiogenesis and invasion inhibition

The anti-angiogenic effect of curcumin has been demonstrated in a variety of tumor cells. Tumor growth, especially in tumors with malignant behavior, where continuous blood supply is the common denominator, tumor vasculature is widely used as a potential target for the treatment of various cancers (25). In studies as early as 2006, it was noted that curcumin was able to inhibit NF-κB-induced VEGF expression, which led to a reduction in angiogenesis (26). In addition, curcumin treatment may lead to a reduction in the expression of MMP-9, an enzyme that degrades the extracellular matrix and is therefore an important marker of tumor invasion and metastasis (27). Several subsequent studies have also indicated that curcumin may inhibit tumor angiogenesis and tumor invasion and metastasis in other tumors such as liver cancer and glioma (28, 29).

In 2012, Shan et al. showed that curcumin may inhibit the growth of PAs by inhibiting the synthesis of HIF-1α and the release of VEGFA in PAs (19). These two key components are involved in tumor neovascularization through angiogenesis, and this inhibitory effect of curcumin was demonstrated in AtT20 cells in mice and GH3 cells in rats. In our previous study, we also found that PA cells were able to induce monocyte osteoclast differentiation and promote bone invasion in a paracrine manner by activating the PKCθ/NF-κB/IL-1β pathway, which also provides a theoretical basis for the inhibition of angiogenesis and invasion of PAs by curcumin (30).

## 3 Regulation of hormone secretion and metabolic homeostasis

The secretion of hormones is accomplished by different glands in the body, affecting a variety of physiological functions, and abnormalities in their secretion can also cause a variety of pathological manifestations. Curcumin is one of the most widely researched drugs for the treatment of endocrine disorders and has unique antioxidant and anti-inflammatory properties. The use of curcumin can lead to significant changes in the levels of many hormones in the body. Polycystic ovary syndrome is a common condition affecting women of childbearing age that can manifest with marked inappropriate gonadotropin secretion and hyperandrogenemia. The disease is strongly associated with oxidative stress. Treatment with curcumin leads to downregulation of the inflammatory state through activation of Nrf 2, IL-SIRT 1 and PGC-1α activation (31). Dehydroepiandrosterone levels were significantly reduced while patients’ estradiol levels remained stable (32). It has been noted in several studies that curcumin-treated patients with type 2 diabetes experienced a significant decrease in serum leptin levels and significant weight loss (33, 34). In fact as early as 2006, the anti-inflammatory effect of curcumin, perhaps effective in the treatment of obesity, was noted in a study by Matchanickal et al (35). On the other hand, curcumin’s effectiveness in treating thyroid dysfunction has been mentioned in many studies. In some models of thyroid function impairment caused by lithium or sodium fluoride exposure, the addition of curcumin significantly elevated TSH secretion levels in rats and improved the symptoms of thyroid dysfunction. In cell lines of papillary thyroid carcinoma, curcumin also effectively inhibited the survival and invasive behavior of tumor cells, and these effects might be related to endoplasmic reticulum stress and cell autophagy.

The key pathological feature of FPAs is excessive secretion of anterior pituitary hormones, leading to associated metabolic abnormalities and clinical manifestations. Bangaru et al.’s study demonstrated that curcumin significantly reduces the secretion of adrenocorticotropic hormone (ACTH) in PA cells, and curcumin exhibits a relatively pronounced concentration-dependent inhibitory effect on ACTH secretion in AtT20 cells (18). This effect may be related to the inhibition of NF-κB transcriptional activity, thereby reducing the expression of its downstream target genes (such as Bcl-xL). In studies using GH3 and MMQ cells, high concentrations of curcumin may also inhibit the excessive secretion of prolactin and growth hormone (16). Although there are minor differences in the doses and patterns of curcumin’s inhibitory effects on hormone secretion across these studies, this does not preclude the hypothesis that such effects indeed exist. The molecular mechanisms underlying these hormone-inhibitory effects have not been fully elucidated, but these phenomena suggest that curcumin may possess objective therapeutic potential in FPAs.

## 4 Therapeutic effects in synergy with drugs

In previous studies, curcumin in many cases acted as a synergistic existing drug to act as an anti-tumor agent. Most of the current cancer treatments recommend combination chemotherapy, and the main advantages utilized may be to reduce the chances of tumor resistance, improve the effective outcome as well as reduce the drug dose. For example, Sudakaran et al. proposed that the combination of curcumin and polyethylene significantly enhances the effect of elastic polyethylene, producing stronger tumor cell inhibition when used alone, for breast cancer treatment (36). In prostate cancer, the combination of low concentrations of curcumin and low concentrations of α-tomatine synergistically inhibited PC-3 cell growth and induced apoptosis, inhibited NF-κB activity and potentially reduced the expression of its downstream gene, Bcl-2, in cells (37). In lung cancer, *in vivo* studies of curcumin and quercetin may synergistically induce apoptosis in lung cancer cells, with better efficacy than the treatment group alone (38). The synergistic anti-tumor effect of curcumin was demonstrated to varying degrees in a variety of tumor cells.

The drug synergistic effect of curcumin in PA cells has been mentioned in previous studies. For prolactin adenomas, dopamine receptor agonists(DAs) are currently the first line of therapy (2, 39). Interestingly, studies suggest that curcumin may potentiate bromocriptine-mediated inhibition of MMQ cell proliferation. Miller et al. treated MMQ cells with bromocriptine in the presence of 5 μ M curcumin and showed that this dose of curcumin by itself did not have a significant growth inhibitory effect on MMQ cell proliferation; however, when MMQ cells were exposed to bromocriptine in the presence of curcumin, even with a lower dose of bromo cryptamine, significant inhibition of cell proliferation was detected (17). In a study by Tang et al. it was proposed that curcumin may sensitize prolactinoma cells to bromocriptine by activating ERK/EGR1 and inhibiting the Akt/GSK-3β signaling pathway (40). On the other hand, the current study suggests that there are abnormalities in miRNA expression in DAs-resistant patients (39). It has been proposed in previous studies that curcumin can affect autophagy in prolactinoma cells by upregulating miR-206 (10). In ovarian cancer studies, curcumin-specific regulation of autophagy by miRNA targeting ATG5 has been validated (41). A study by Wu et al. demonstrated that miR-93 was upregulated in drug-resistant prolactinoma cells and mediated DA resistance through downregulation of autophagy targeting ATG7 (42). These studies provide new research directions for curcumin synergistic therapy.

Temozolomide (TMZ), as an important treatment for aggressive and refractory PAs, has been classified by the European Society of Endocrinology (ESE) as the first-line treatment for aggressive PAs and pituitary carcinomas, but the objective remission rate of its single-agent treatment is only 37% (43). Currently, the regimen of temozolomide combined with radiotherapy is mostly used to improve the remission rate. However, the problems of hypopituitarism, optic nerve damage, and increased intracranial pressure associated with radiotherapy cannot be ignored (44). Research in other tumor areas, especially in intracranial tumors, curcumin as a combination of temozolomide is relatively promising. It is generally believed that curcumin can sensitize glioma cells to various chemotherapeutic regimens by down-regulating Bcl-2 and IAP family members, as well as DNA repair enzymes (e.g., MGMT, DNA-PK, and ERCC-1) (11). In the published clinical study by Liang et al, curcumin may play an important role in the inhibition of temozolomide resistance with low toxicity and side effects by injecting curcumin along with temozolomide through a carrier into the tumor cavity of gliomas (45). Based on these molecular mechanisms and clinical findings, curcumin may be able to potentiate temozolomide in aggressive pituitary tumors as well.

Therapeutic potential of curcumin for pituitary-related metabolic disorders

One of the most important reasons for curcumin’s potential as a therapeutic agent for PAs is that in addition to its current highly promising research on anti-tumor effects, there is also an endocrine metabolism therapeutic component for which a great deal of research already exists. This may have a multiplier effect for PAs, a tumor that may cause severe endocrine symptoms. It has already been mentioned that curcumin may alleviate FPA symptoms by inhibiting the overproduction of PA-related hormones (GH, PRL, ACTH, etc.) (16, 17). The overproduction of these anterior pituitary hormones can itself lead to more associated metabolic abnormalities.

Growth hormone, as a potent glucagon, may interfere with glucose metabolic homeostasis through multiple pathways.GH overproduction persistently activates the JAK2-STAT5 signaling pathway in liver and muscle tissue, leading to abnormal phosphorylation of insulin receptor substrate (IRS) and impeding insulin signaling (46). In the liver, GH increases hepatic glucose output by stimulating the expression of key enzymes of gluconeogenesis (phosphoenolpyruvate carboxykinase, glucose-6-phosphatase), and in muscle and adipose tissues, it decreases glucose uptake by inhibiting GLUT4 translocation. Studies have shown that insulin sensitivity in patients with acromegaly can be reduced by more than 40% compared with normal subjects, which is the main mechanism by which GH adenomas cause hyperglycemia (47). On the other hand, early GH elevation produces a pro-proliferative effect on β-cells, which is manifested by a compensatory increase in insulin secretion and the formation of hyperinsulinemia. However, long-term GH overstimulation will lead to β-cell endoplasmic reticulum stress enhancement and de-differentiation phenomenon, and eventually trigger β-cell failure (48). In addition, GH also promotes lipolysis and increases the level of free fatty acids (FFA), which further impairs β-cell function through lipotoxicity and competitively inhibits glucose oxidation in muscle tissue through the Randle cycle, exacerbating insulin resistance (49). Patients with large limbs are often associated with visceral fat accumulation, thus creating a vicious circle. Currently, surgical treatment is the fundamental means to relieve growth hormone overproduction (50). In the past, our center also succeeded in normalizing blood glucose in patients with GH adenoma through satisfactory tumor resection, which shows that early surgical intervention has a definite alleviating effect on diabetes mellitus caused by pituitary growth hormone adenoma. For those GH adenomas with strong aggressiveness and high recurrence rate, the drug treatment plan needs to be formulated more according to individual differences. Currently, the preferred drug is Somatostatin analogs (SSA), which inhibits GH secretion by activating the SSTR2/SSTR5 receptors of pituitary tumor cells (51).

Current insulin sensitizers (metformin, etc.) are the mainstay of the treatment of diabetes with insulin resistance, however, the long-term clinical use of these drugs is associated with several harmful side effects. The study of new drugs that can avoid these side effects is currently a hot topic of research. It has been shown that curcumin, on the one hand, attenuates insulin resistance by decreasing IRS-1 serine phosphorylation and increasing IRS-1 tyrosine phosphorylation in rat skeletal muscle (52). On the other hand, curcumin can reduce hepatic glucose output by activating the AMPK pathway to enhance glucose uptake in skeletal muscle, which in turn inhibits key enzymes of gluconeogenesis (e.g., PEPCK) and improves glucose tolerance abnormality in patients with PAs (53). In addition, curcumin reduced TNF-α and C-reactive protein (CRP) levels and inhibited extracellular kinase 1/2 (ERK 1/2) and p38 expression in rat skeletal muscle (54). This study emphasizes that curcumin possesses significant antioxidant and anti-inflammatory properties and has considerable potential in the treatment of glucose intolerance and insulin resistance. Also in other studies, curcumin induced the production of other pro-inflammatory cytokines (IL-6, etc.) and the activation of antioxidant factors (Nrf-2, etc.) in adipose tissue and liver, and these inflammatory macrophages promoted insulin resistance (55). Ahmed et al. showed that curcumin could reduce the metabolic syndrome in the context of hyperglycemia-induced vascular endothelial injury by interfering with the formation of AGEs and AGE-induced vascular injury. vascular injury to attenuate excessive vasoconstriction in metabolic syndrome (56). Free radical scavenging and direct vasodilatory activity may also contribute to the favorable vascular effects of curcumin. Curcumin has also been shown to play an active role in insulin resistance in a number of other diseases. polycystic ovary syndrome (PCOS) patients are mostly characterized by insulin resistance. In a recent study, curcumin showed synergistic effects with metformin in improving insulin resistance in PCOS patients. Curcumin may attenuate insulin resistance and maintain the integrity of pancreatic islets by increasing the levels of PI3K/Akt/mTOR (32). This suggests that if curcumin is applied to pituitary GH adenomas, it may inhibit tumor growth and hormone secretion, while at the same time play a protective role against target organ damage caused by abnormal glucose metabolism.

Cortisolism due to ACTH adenomas can likewise trigger abnormalities in glycolipid metabolism and oxidative stress injury in humans. In a study by Bangaru et al, it was noted that curcumin alleviated the above damage in mouse pituitary adrenocorticotropic hormone cytoma AtT20 cells by reducing the release of inflammatory factors (e.g., IL-6, TNF-α) through inhibition of the NF-κB signaling pathway by a mechanism similar to that in GH adenomas (18). As for the reduction of bone mineral density induced by PRL, ACTH and GH excess, Cirano et al. pointed out that curcumin can maintain bone metabolism by inhibiting the RANKL/OPG pathway to reduce osteoclast differentiation, improve the level of β-catenin, and promote the activity of osteoblasts. This suggests that curcumin may play a complex role not only in GH adenomas, but also in other FPAs with more efficient therapeutic effects.

## 5 Challenges to the current clinical application of curcumin

### 5.1 Poor penetration across the blood-brain barrier

PAs originate in the saddle region, and the selection of an appropriate method to deliver curcumin across the blood-brain barrier is a prerequisite for the drug to work. The bottleneck for curcumin to penetrate the blood-brain barrier stems from its physicochemical properties and metabolic defects; its water solubility is only 0.012 mg/mL, which makes it difficult to penetrate the blood-brain barrier by passive diffusion. On the other hand, P-glycoprotein (P-gp) and other efflux pumps on the blood-brain barrier will actively expel curcumin from the brain tissue, which will further reduce the accumulation of curcumin in the brain (57).

Research in nanotechnology (brain-targeted peptide modification, lipid emulsions) and novel delivery systems such as supramolecular carriers and slow-release hydrogels has now led to breakthroughs (58). Significant enhancement of blood-brain barrier penetration by targeting transferrin receptor (TfR). Studies have shown that nanoparticles loaded with curcumin and S1-peptide successfully reduced Aβ plaques and neuroinflammation in an Alzheimer’s disease (AD) model (58). Further, following the study of curcumin in gliomas, targeted delivery of curcumin could be considered using, for example, similar transferrin-modified nanovesicles. The characteristics of existing nanocarriers are summarized in detail in the review by Hussain et al (59). We have created **Table 2** based on that review. In the future, there is a greater need to focus research on the precise delivery of curcumin as well as individualized dose adjustment for its use.

**Table 2:** Advantages and Limitations of existing curcumin nanocarriers.

Nanocarrier type	Advantages	Limitations
Polymeric nanoparticles	Strong targeting, sustained release	Complex synthesis, scalability challenges
Liposomes	High biocompatibility, ease of modification	Limited stability, drug leakage
Polymeric micelles	High drug loading, combinable with PDT	Rapid clearance *in vivo*
Nanogels	High encapsulation efficiency, transdermal potential	Low mechanical strength, prone to degradation
Metal nanoparticles	Multifunctionality	Potential metal toxicity requires careful evaluation

### 5.2 Low bioavailability of curcumin

Curcumin is highly lipid-soluble and poorly water-soluble, making it difficult to be effectively absorbed through the intestinal tract. More than 90% of orally administered curcumin is metabolized in the liver to inactive, inert glucuronide conjugates with a short molecular half-life. High-dose administration has been attempted in previous studies, but the attendant hepatotoxicity problems are impossible to ignore. High doses of curcumin may lead to tolerance problems beyond the patient’s control, such as experiencing allergies, gastrointestinal disturbances such as nausea, diarrhea, or stomach upset (7). In addition, curcumin may affect blood clotting. Numerous studies have shown that curcumin has anticoagulant and antiplatelet properties that may increase the risk of bleeding when anticoagulants (e.g., warfarin) are applied, and this is a possible pitfall to be aware of with high doses of curcumin (60).

Currently there are three main categories of approaches regarding the enhancement of curcumin bioavailability. The first category is through the association of other catabolic antagonists (e.g., desmethoxycurcumin) to reduce curcumin metabolism or potentiators (e.g., piperine, etc.) to increase the area under the curve (AUC) at the time of pharmacological administration (61). The second category is through the use of novel delivery systems (e.g., the nano-formulations mentioned above) that not only allow curcumin to cross the blood-brain barrier, but also improve its solubility and delay metabolism. The third category is the direct structural modification of curcumin, such as the incorporation of hydrophilic structures (hydroxyethyl starch, etc.), in order to improve the water solubility and antioxidant activity of the medication (61).

### 5.3 Little research in the field of PAs

The molecular mechanism of curcumin in PAs is not well studied and few clinical studies have been reported. The current direction of research is mostly based on extrapolation of findings from other tumors such as colon cancer, prostate cancer and breast cancer. The molecular mechanism, efficacy and safety of curcumin in the treatment of PAs need to be explored in more studies.

## 6 Conclusion

Curcumin, with its multi-targeted anti-tumor properties, has shown potential value in the treatment of PAs. However, its clinical application is limited by its low bioavailability and insufficient targeting. Curcumin not only inhibits the progression of pituitary adenomas through multi-targets, but also systematically improves the metabolic complications caused by hormonal abnormalities, which has the potential of “one drug, multiple effects”. In the future, it is necessary to combine the novel delivery technology and mechanism research to promote its translation from laboratory to clinic. We need to combine targeted delivery technology and metabolomics research to promote its application in the comprehensive treatment of pituitary adenomas.
